# Adherence to the Mediterranean diet and allergic diseases in Korean adults: KNHANES 2013–2016

**DOI:** 10.3389/fnut.2025.1563251

**Published:** 2025-04-07

**Authors:** Kaiyue Tan, Nanren Sun, Jiaojiao Chen, Jiaqi Long, Wenzhe Feng, Xiaojie Zhang, Zhimin Tan

**Affiliations:** ^1^The First Clinical College of Shandong University of Traditional Chinese Medicine, Jinan, Shandong, China; ^2^Department of Dermatology, Affiliated Hospital of Shandong University of Traditional Chinese Medicine, Jinan, Shandong, China; ^3^Department of Otolaryngology, Affiliated Hospital of Shandong University of Traditional Chinese Medicine, Jinan, Shandong, China

**Keywords:** allergic diseases, Mediterranean diet, KNHANES, mMED, gender differences

## Abstract

**Background:**

The prevalence of allergic diseases [e.g., asthma, allergic rhinitis (AR), atopic dermatitis (AD)] has increased significantly in recent years, which is coincides with a shift in modern eating habits. The Mediterranean diet, due to its anti-inflammatory properties, may be beneficial in the prevention of allergic diseases. However, its effects on allergic diseases have not been sufficiently studied. We investigated the relationship between adherence to the Mediterranean diet and allergic diseases.

**Methods:**

This study analyzed the relationship between adherence to the Mediterranean diet (using the modified Mediterranean diet score, mMED) and atopic dermatitis, asthma, and allergic rhinitis in 12,080 participants using data from the 2013–2016 Korean National Health and Nutrition Examination Survey (KNHANES). Multiple logistic regression analyses were used to control for confounding factors such as age, gender, education level, income, and lifestyle.

**Results:**

In multivariable adjusted models, participants with higher mMED had a significantly lower risk of developing AD (OR 0.57; 95% CI, 0.36–0.92; *p* trend = 0.0201). When stratified by sex, this risk reduction was more significant in females (OR 0.50; 95% CI, 0.27–0.96; *p* trend <0.05). Across mMED components, fish and peanut intake were negatively associated with the occurrence of AD and AR (OR 0.55; 95% CI, 0.40–0.76; *p* trend <0.05, OR 0.75; 95% CI, 0.65–0.87; *p* trend <0.05). There was no significant association between asthma and AR and mMED scores.

**Conclusion:**

High adherence to the Mediterranean diet is associated with a lower prevalence of atopic dermatitis, especially in women. Fish and peanut intake have an important protective role against atopic diseases.

## 1 Introduction

In recent decades, the prevalence of allergic diseases, such as asthma, allergic rhinitis (AR), and atopic dermatitis (AD), has risen sharply. Research (1) have shown that this trend coincides with a shift in modern eating habits. Modern diets are usually based on ultra-processed foods and low consumption of fruits and vegetables, which leads to insufficient intake of dietary fiber, yet excessive intake of sugar, saturated fats, and Omega-6 unsaturated fats (2). This diet is strongly associated with an increase in allergic diseases, as the lack of dietary fiber, as well as the intake of excess sugar, Omega-6 unsaturated fats, and saturated fats may increase inflammation in the body, which may trigger or exacerbate these diseases (3–5).

In contrast, the Mediterranean diet is widely regarded as a healthier eating pattern (6, 7). It is defined by a high intake of fruits, vegetables, whole grains, and moderate amounts of fish and poultry, with limited consumption of red meat and dairy. Emphasizing high-fiber foods, healthy fats, and minimal processed foods, the Mediterranean diet is abundant in antioxidants, polyunsaturated fats, and essential vitamins. It is also relatively low in saturated fats (8), which contributes to its strong anti-inflammatory benefits. As a result, long-term adherence to the Mediterranean diet not only enhances overall health but also lowers the risk of chronic conditions, including allergic diseases (9–11).

Several studies suggest that the Mediterranean diet offers a protective effect against atopic dermatitis (AD) (12). Although the Mediterranean diet shows promise in managing AD, its effects on asthma and allergic rhinitis (AR) appear more complex. Some research indicates that the Mediterranean diet may help protect against asthma, potentially due to its anti-inflammatory properties and its ability to modulate the immune system (13). However, findings regarding the Mediterranean diet’s impact on AR have been less consistent. One study concluded that the Mediterranean diet had little impact on improving AR, possibly due to variations in the immune mechanisms that drive AR compared to those involved in skin inflammation (14). These varied results highlight the need for more research to better understand the relationship between the Mediterranean diet and allergic diseases. The aim of this study was to explore the relationship between adherence to the Mediterranean diet and allergic diseases in Korean adults using data from the 2013–2016 Korean National Health and Nutrition Examination Survey (KNHANES).

## 2 Methods

### 2.1 Design and data collection

**Figure 1:**
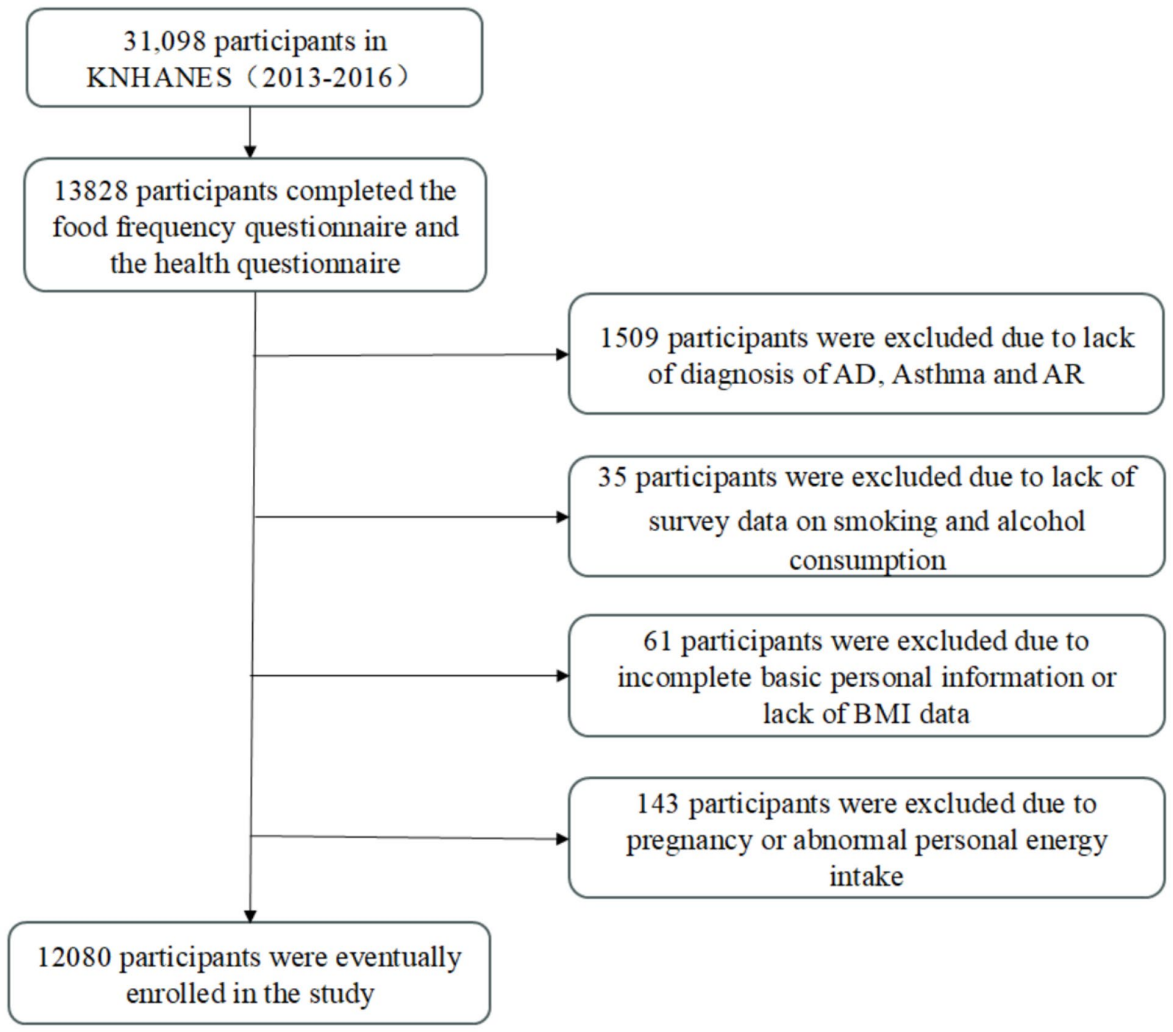
Study participant flowchart.

This study utilized data from the Korea National Health and Nutrition Examination Survey (KNHANES) VI (2013–2015) and VII (2016), covering a 4-year period between 2013 and 2016. The Korea Centers for Disease Control and Prevention (KCDC) administers KNHANES to track trends in health risk factors and their prevalence. The survey includes health screenings, health interviews, and nutritional assessments, all conducted by trained medical professionals and interviewers. The survey was approved by the Institutional Review Board of the KCDC, and all participants provided informed consent. Further details and descriptions of the database are available on the KNHANES website^1^. From the 2013–2016 surveys, 31,098 individuals were sampled, with 13,828 completing the Food Frequency Questionnaire (FFQ). We excluded participants who: did not fully respond to questions about AD, asthma, and AR (*n* = 1,509); did not answer questions on smoking or alcohol consumption (*n* = 35); had incomplete personal information (education level, household income quartiles, occupation, or exercise activity) or missing BMI data (*n* = 61); and those who were pregnant or had abnormal energy intake (EK <500 kcal or EK >6,000 kcal) (*n* = 143). Consequently, 12,080 participants were eligible for inclusion in our study (Figure 1).

### 2.2 Definition of covariates

The following variables were used as covariates: age (years), gender (male or female), residence area (urban or rural), education level (primary school or below, junior high school, high school, and university or above), and body mass index (BMI), calculated as weight (kg) divided by height squared (m^2^). Based on obesity reference values for Asian populations (15), participants were categorized into three groups: underweight (BMI < 18.5), normal (18.5 ≤ BMI < 25), and obese (BMI ≥ 25). Household income was divided into quartiles (low, medium, upper-middle, and upper). Alcohol consumption was classified into drinkers and non-drinkers, while smoking status was divided into non-smokers (*n* ≤ 100 cigarettes) and smokers (*n* > 100 cigarettes). Participants were also categorized by strength exercise frequency: those who did not perform strength exercises (*n* < 2 days per week) and those who did (*n* ≥ 2 days per week).

### 2.3 Assessment of modified Mediterranean diet scores

To account for the differences in dietary habits between Asians and Europeans, we used the modified Mediterranean Diet score (mMED), developed by Kim and Je (16), to measure adherence to the Mediterranean diet in the Korean population and to adjust Korean dietary patterns accordingly. Dietary intake was evaluated using the semi-quantitative Food Frequency Questionnaire (FFQ) from the Korean National Health and Nutrition Examination Survey (KNHANES) conducted between 2013 and 2016. The FFQ contains 112 items designed to assess the average food intake and frequency of consumption over the previous year. The frequency of food intake was categorized into nine options, ranging from “hardly ever” to “three times a day.” Average food intake was divided into three categories: “half the standard intake,” “equal to the standard intake,” and “1.5 or 2 times the standard intake.” Daily intake for each food item was calculated by multiplying the frequency of consumption by the average intake.

The modified Mediterranean Diet score (mMED) classified 65 foods into nine categories: vegetables, legumes, fruits, whole grains, red or processed meats, white meats, fish or peanuts, dairy products, and alcohol. mMED was calculated using the median daily intake for each food group as the threshold. The total mMED score ranged from 0 to 9, with each food group scored either 0 or 1. Participants received one point for consuming more than the median intake for vegetables, legumes, fruits, whole grains, white meat, fish or peanuts, and dairy products. For red or processed meat, given Korea’s relatively low consumption of red meat, one point was awarded if intake was below the 75th percentile. Ethanol intake from alcoholic beverages was calculated using the 8th revision of the Korean Food Composition Table by the Korean Nutrition Society, with one point awarded for a daily intake of 0–15 grams of ethanol. If intake exceeded this, zero points were given. For statistical analysis, mMED was divided into four categories: 0–2, 3–4, 5–6, and 7–9 points.

### 2.4 Assessment of allergic diseases

The presence of allergic diseases was determined through a health interview survey. For example, atopic dermatitis (AD) was identified if participants answered “yes” to the question, “Have you been diagnosed with AD by a doctor?.” The same approach was used to diagnose asthma and allergic rhinitis (AR).

### 2.5 Statistical analyses

Statistical analyses were conducted using the PROC SURVEY procedure in SAS software (version 9.4, SAS Institute Inc., Cary, NC, USA), with survey weights applied to account for the complex sampling design of KNHANES. Differences in participant characteristics were assessed using chi-square tests. Multinomial logistic regression models were employed to analyze the association between the modified Mediterranean Diet score (mMED) and the three allergic diseases (AR, AD, and asthma). Two models were used for covariate adjustment: Model 1 was adjusted for age and gender only, while Model 2 was a multivariate model that controlled for education level, household income, occupation, lifestyle factors (alcohol consumption, smoking, and exercise), BMI, and total energy intake. Stratified analyses were also performed to examine the relationship between mMED and the three allergic diseases by gender. The results from multinomial logistic regression are presented as odds ratios (OR) with 95% confidence intervals (CI). All tests were two-sided, and a *p*-value <0.05 was considered statistically significant.

## 3 Results

### 3.1 mMED association analysis with lifestyle, socioeconomic status and energy intake

Table 1 presents the characteristics of study participants based on quartiles of the modified Mediterranean Diet score (mMED). The data indicated that higher mMED scores were significantly associated with older age, non-smoking and non-alcoholic lifestyles, higher levels of education and household income, more frequent strength training activities, and lower BMI. Additionally, higher mMED scores were positively correlated with greater total energy intake.

**Table 1:** Demographic and lifestyle characteristics of study participants in four groups of modified alternative Mediterranean diet scores, KNHANES 2013–2016 (*n* = 12,080).

mMED group	T1 (*n* = 908)	T2 (*n* = 4,171)	T3 (*n* = 4,724)	T4 (*n* = 2,277)	*p*-value
Covariate					
Age (years)	38.33 ± 0.49	39.58 ± 0.23	41.75 ± 0.21	44.41 ± 0.30	**< 0.0001**
Sex					**< 0.0001**
Men	60.24 (0.25)	50.41 (0.43)	46.11 (0.42)	37.34 (0.27)	
Women	39.76 (0.18)	49.59 (0.38)	53.89 (0.39)	62.66 (0.29)	
Residential area					0.5820
Urban	85.41 (0.28)	84.47 (0.49)	85.08 (0.49)	85.90 (0.36)	
Rural	14.59 (0.11)	15.53 (0.24)	14.92 (0.24)	14.10 (0.15)	
Edu					**< 0.0001**
Primary or below	9.88 (0.10)	8.26 (0.16)	7.12 (0.15)	5.73 (0.09)	
Middle school	9.30 (0.09)	6.86 (0.14)	6.84 (0.15)	9.33 (0.12)	
High school	43.63 (0.21)	42.46 (0.39)	39.43 (0.38)	42.02 (0.27)	
College or above	37.19 (0.19)	42.42 (0.38)	46.61 (0.40)	42.92 (0.27)	
Occupation					**0.0002**
Non-manual labor	73.76 (0.26)	77.11 (0.47)	78.22 (0.47)	81.31 (0.35)	
Manual labor	26.24 (0.16)	22.89 (0.29)	21.78 (0.29)	18.69 (0.18)	
Household income					**< 0.0001**
Q1	14.25 (0.12)	9.49 (0.19)	8.20 (0.18)	6.41 (0.11)	
Q2	24.32 (0.15)	26.75 (0.32)	22.68 (0.30)	21.40 (0.19)	
Q3	33.71 (0.19)	33.04 (0.34)	32.51 (0.34)	30.53 (0.23)	
Q4	27.72 (0.16)	30.72 (0.33)	36.61 (0.36)	41.66 (0.26)	
Smoking status					**< 0.0001**
No	44.68 (0.20)	57.71 (0.42)	64.34 (0.44)	73.68 (0.33)	
Yes	55.14 (0.24)	42.29 (0.40)	35.66 (0.37)	26.32 (0.22)	
Drinking status					**< 0.0001**
No	4.15 (0.05)	5.19 (0.14)	7.02 (0.16)	9.36 (0.11)	
Yes	95.85 (0.30)	94.81 (0.50)	92.98 (0.50)	90.64 (0.38)	
Weight training					**< 0.0001**
No	83.62 (0.28)	80.96 (0.48)	75.34 (0.46)	72.72 (0.33)	
Yes	16.38 (0.13)	19.04 (0.28)	24.66 (0.32)	27.28 (0.22)	
BMI					**0.0055**
Lean	4.97 (0.08)	5.64 (0.16)	4.20 (0.13)	4.22 (0.08)	
Normal	61.31 (0.24)	62.95 (0.44)	62.96 (0.44)	66.95 (0.32)	
Obesity	33.72 (0.18)	31.41 (0.33)	32.84 (0.35)	28.83 (0.22)	
Energy intake (kcal/day)	1782.65 (29.98)	1889.57 (13.60)	2209.99 (14.16)	2463.82 (20.88)	**< 0.0001**
Carbohydrates (%kcal/day)	60.45 (0.45)	63.34 (0.18)	63.30 (0.14)	64.05 (0.18)	**< 0.0001**
Protein (%kcal/day)	11.32 (0.10)	12.60 (0.04)	13.49 (0.03)	14.21 (0.05)	**< 0.0001**
Fats (%kcal/day)	16.04 (0.26)	17.44 (0.11)	18.74 (0.09)	19.40 (0.12)	**< 0.0001**

T1 (0–2 points), T2 (3–4 points), T3 (5–6 points), T4 (7–9 points). All results are weighted and presented as means and standard errors (SE) for continuous variables and percentages and SE for categorical variables. Bold values are defined as significant at *p* values < 0.05.

### 3.2 Association of mMED with allergic diseases

Table 2 shows the cross-sectional associations between the modified Mediterranean Diet score (mMED) and AD, asthma, and AR. Before adjusting for covariates, participants in the highest mMED quartile had a 46% lower risk of developing AD compared to those in the lowest quartile (OR 0.54, 95% CI 0.35–0.84, *p* trend = 0.0059). This inverse association became non-significant after adjusting for age and sex (OR 0.69, 95% CI 0.44–1.09, *p* trend = 0.1094), but it reappeared after adjusting for all covariates (OR 0.57, 95% CI 0.36–0.92, *p* trend = 0.0201). It is important to note that no significant associations were found between mMED and asthma or AR.

**Table 2:** Ratios and 95% confidence intervals for multivariate logistic regression of the association between modified Mediterranean diet score (mMED) and allergic diseases.

	Mediterranean diet score						
	0–2	3–4		5–6		7–9	
	ORs	ORs (95% CIs)	*p*-value	ORs (95% CIs)	*p*-value	ORs (95% CIs)	*p*-value
Model 1a*	1.0 (ref)	0.96 (0.67–1.41)	0.8147	0.78 (0.54–1.15)	0.1928	**0.54 (0.35–0.84)**	**0.0059**
Model 2a*	1.0 (ref)	1.01 (0.70–1.49)	0.9633	0.89 (0.62–1.32)	0.5564	0.69 (0.44–1.09)	0.1094
Model 3a*	1.0 (ref)	0.98 (0.67–1.44)	0.9251	0.80 (0.54–1.19)	0.2753	**0.57 (0.36–0.92)**	**0.0201**
Model 1b*	1.0 (ref)	1.21 (0.77–2.02)	0.4301	1.02 (0.64–1.70)	0.9387	0.91 (0.55–1.59)	0.7444
Model 2b*	1.0 (ref)	1.18 (0.74–1.96)	0.5115	0.98 (0.61–1.63)	0.9185	0.86 (0.51–1.50)	0.5689
Model 3b*	1.0 (ref)	1.20 (0.74–1.95)	0.4674	0.00 (0.60–1.64)	0.9839	0.88 (0.50–1.56)	0.6671
Model1c*	1.0 (ref)	1.01 (0.83–1.23)	0.9018	1.07 (0.88–1.30)	0.4854	0.95 (0.77–1.17)	0.6069
Model 2c*	1.0 (ref)	1.00 (0.83–1.23)	0.9667	1.10 (0.91–1.34)	0.3432	1.00 (0.81–1.25)	0.9676
Model 3c*	1.0 (ref)	0.99 (0.81–1.21)	0.9523	1.09 (0.89–0.33)	0.4156	0.99 (0.79–1.24)	0.9239

Model 1 is unadjusted. Model 2 is sex and age adjusted. Model 3 was multivariate adjusted. a, Atopic dermatitis model; b, Asthma model; c, Allergic rhinitis model. Each Modified Mediterranean Diet score group was compared with the 0–2 subgroups, Bold values are defined as significant at *p* values < 0.05.

### 3.3 Association of components of mMED with allergic diseases

**Figure 2:**
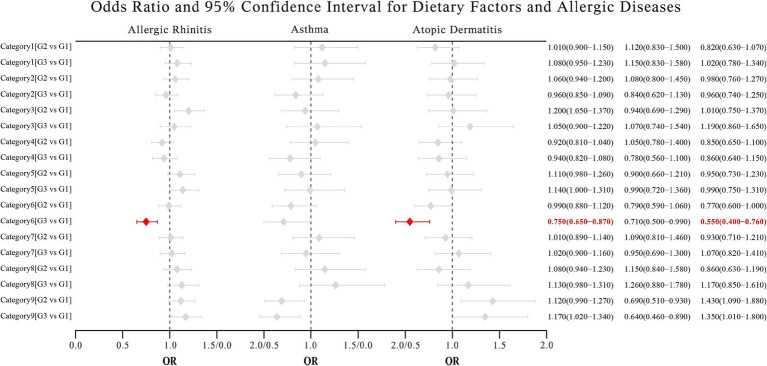
Association of components of the alternative Mediterranean diet score with allergic diseases. Categories 1–9: Vegetables, legumes, fruits, whole grains, red or processed meat, white meat, fish or peanuts, dairy products and alcohol. G1-3: Tertile grouping of daily intake. We corrected the results for False Discovery Rate (FDR) and a *p*-value <0.05 was considered significant.

Figure 2 illustrates the associations between individual components of mMED and allergic diseases, after adjusting for covariates. Analysis of individual mMED components revealed that fish and peanut intake were negatively associated with AD and AR, while other components showed no significant associations with these conditions. Furthermore, no significant associations were identified between mMED components and asthma.

### 3.4 Gender differences in the effect of adherence to the Mediterranean diet on allergic diseases

Table 3 presents the association between mMED, covariates, and allergic diseases based on sex-stratified analyses. For AD, the analysis showed that in men, older age and smoking were protective factors, while in women, older age and higher mMED significantly reduced the risk of AD. Conversely, lower mMED increased the risk, a finding confirmed by bidirectional validation. In the asthma analysis, older age was protective in men, while higher education levels provided protection against asthma in women. In the stratified analysis of AR, protective factors for men included older age, physical labor, smoking, and higher BMI. For women, protective factors were older age, physical labor, and higher energy intake. In contrast, alcohol consumption and increased strength training were identified as risk factors for women. These results suggest that gender, age, and lifestyle factors play different roles in the development and progression of allergic diseases. Notably, significant gender differences were observed in the association between mMED and AD.

**Table 3:** Ratios and 95% confidence intervals of multivariate logistic regressions of gender differences in the effect of adherence to the Mediterranean diet on allergic diseases.

	Atopic dermatitis		Asthma		Atopic rhinitis	
	Men	Women	Men	Women	Men	Women
mMED [G2 VS G1]	1.17 (0.63–2.18)	0.83 (0.48–1.45)	1.95 (0.72–5.27)	0.88 (0.47–1.64)	1.13 (0.81–1.58)	0.87 (0.65–1.15)
mMED [G3 VS G1]	1.31 (0.70–2.45)	0.64 (0.37–1.11)	1.72 (0.63–4.66)	0.77 (0.41–1.46)	1.10 (0.79–1.54)	1.06 (0.79–1.40)
mMED [G4 VS G1]	0.97 (0.45–2.09)	**0.50 (0.27–0.96)**	0.83 (0.24–2.94)	0.72 (0.37–1.40)	0.96 (0.64–1.42)	0.97 (0.72–1.32)
Age [G2 VS G1]	**0.29 (0.18–0.45)**	**0.15 (0.09–0.24)**	**0.42 (0.25–0.69)**	0.81 (0.56–1.17)	**0.53 (0.43–0.66)**	**0.57 (0.48–0.67)**
Residential area [G2 VS G1]	0.82 (0.50–1.37)	0.92 (0.60–1.41)	0.92 (0.51–1.65)	1.24 (0.79–1.96)	0.77 (0.58–1.01)	1.03 (0.84–1.26)
Household income	0.97 (0.81–1.18)	0.91 (0.77–1.07)	1.02 (0.81–1.28)	1.01 (0.85–1.21)	0.99 (0.89–1.10)	0.96 (0.89–1.03)
Edu [G2 VS G1]	0.77 (0.25–2.38)	1.01 (0.39–2.66)	0.70 (0.19–2.54)	0.80 (0.42–1.50)	1.50 (0.78–2.86)	0.97 (0.68–1.38)
Edu [G3 VS G1]	0.65 (0.25–1.67)	0.54 (0.28–1.44)	0.51 (0.16–1.61)	**0.42 (0.26–0.68)**	1.24 (0.72–2.13)	0.98 (0.72–1.32)
Edu [G4VS G1]	0.41 (0.15–1.10)	0.58 (0.25–1.38)	0.61 (0.19–1.98)	**0.41 (0.24–0.71)**	1.50 (0.86–2.62)	1.08 (0.78–1.50)
Occupation [G2 VS G1]	0.75 (0.48–1.19)	0.73 (0.39–1.37)	0.96 (0.57–1.63)	0.63 (0.36–1.09)	**0.75 (0.60–0.95)**	**0.72 (0.57–0.92)**
Smoking status [G2 VS G1]	**0.71 (0.50–1.00)**	1.01 (0.62–1.63)	0.93 (0.57–1.50)	1.52 (0.94–2.47)	**0.81 (0.67–0.99)**	1.16 (0.93–1.46)
Drinking status [G2 VS G1]	1.32 (0.44–3.99)	1.26 (0.67–2.38)	0.78 (0.23–2.71)	0.93 (0.55–1.57)	1.03 (0.60–1.75)	**1.31 (1.01–1.70)**
Weight training [G2 VS G1]	1.31 (0.02–1.85)	1.10 (0.74–1.65)	1.60 (0.98–2.61)	1.31 (0.86–1.98)	0.99 (0.81–1.21)	**1.25 (1.05–1.50)**
BMI [G1 VS G2]	0.62 (0.20–1.88)	0.95 (0.53–1.68)	2.16 (0.81–5.76)	1.49 (0.79–2.79)	0.99 (0.53–1.83)	1.28 (0.99–1.67)
BMI [G3 VS G2]	1.25 (0.89–1.77)	0.75 (0.50–1.11)	1.18 (0.74–1.88)	1.36 (0.92–2.00)	**0.74 (0.61–0.89)**	0.87 (0.73–1.04)
Energy intake	1.10 (0.94–1.29)	**1.24 (1.03–1.49)**	1.09 (0.87–1.38)	0.92 (0.73–1.15)	1.03 (0.94–1.13)	**0.91 (0.83–0.99)**
mMED [G1 VS G4]	1.03 (0.48–2.22)	**1.98 (1.05–3.77)**	1.20 (0.34–4.25)	1.39 (0.71–2.72)	1.05 (0.70–1.56)	1.03 (0.76–1.40)
mMED [G2 VS G4]	1.21 (0.67–2.18)	**1.65 (1.02–2.66)**	2.34 (0.94–5.84)	1.22 (0.77–1.93)	1.18 (0.88–1.59)	0.89 (0.73–1.08)
mMED [G3 VS G4]	1.35 (0.76–2.40)	1.26 (0.78–2.04)	2.06 (0.84–5.08)	1.08 (0.69–1.68)	1.15 (0.86–1.55)	1.09 (0.91–1.30)

Age was divided into two groups according to the median, area of residence was divided into urban and rural areas, occupation was divided into non-manual and manual labor groups, education level was divided into four groups: primary school graduation and below, junior high school graduation, high school graduation and university or above, BMI was divided into three groups: thin (BMI < 18.5), normal (18.5 ≤ BMI < 25) and obese (BMI ≥ 25), household income and energy intake were analyzed as continuous variables. Bold values are defined as significant at *p* values < 0.05.

These results suggest that factors such as gender, age and lifestyle have different influences in the development and progression of allergic diseases, and in particular, there are significant gender differences in the association of mMED with AD.

## 4 Discussion

In this cross-sectional study of Korean adults, higher mMED scores, indicating greater adherence to the Mediterranean diet, were associated with a lower risk of developing atopic dermatitis (AD). Among the nine components of mMED, only fish and peanuts were negatively associated with AD and allergic rhinitis (AR). After stratifying by gender, we identified different protective and risk factors for each disease, with older age emerging as a general protective factor. To our knowledge, this is the first study exploring the relationship between the Mediterranean diet and allergic diseases in an Asian population.

The mechanism behind the Mediterranean diet’s effect on allergic diseases remains unclear, but existing studies suggest that the diet’s high content of antioxidants (e.g., vitamins C, E, and polyphenols) reduces oxidative stress and inflammatory markers, alleviating allergic symptoms and reactions (10, 13, 14, 17). Additionally, the Mediterranean diet’s high dietary fiber content promote a healthy gut microbiota and enhance immune tolerance (18, 19). Healthy fats in the diet inhibit pro-inflammatory cytokine production and improve skin barrier function (20). This suggests that the Mediterranean diet boosts immune system function, increasing the body’s resistance to allergic diseases. By enhancing immune function and reducing bodily inflammation, the Mediterranean diet can lower the frequency and severity of allergic disease episodes (20). The Mediterranean diet is also strongly linked to improved mental health, with a balanced diet helping to lower levels of depression and anxiety, which in turn may alleviate symptoms of allergic diseases (21, 22).

In our analysis of the Mediterranean diet components, we found that fish and peanuts provided the most significant protection against allergic diseases. Studies have shown that fish, particularly those rich in omega-3 fatty acids like salmon and mackerel, possess strong anti-inflammatory properties. Omega-3 fatty acids enhance skin barrier function, reduce skin inflammation, and help lower inflammatory responses in the airways and nasal passages (23, 24). Peanuts, rich in monounsaturated fats and antioxidants, exert immunomodulatory and anti-inflammatory effects by balancing Th1 and Th2 cell responses and inhibiting the production of pro-inflammatory cytokines (25). Additionally, peanut consumption has been linked to a diverse gut microbiota, which strengthens intestinal barrier function and reduces systemic inflammation, thereby lowering the risk of atopic diseases (26).

Our study indicates that strict adherence to the Mediterranean diet is closely linked to a reduced risk of AD, which is considered a key factor in the progression to asthma and AR, a phenomenon commonly referred to as the “atopic march.” Patients with AD typically experience symptoms like dry, itchy skin and rashes during childhood, which can later progress into other allergic conditions such as asthma and AR. Therefore, early prevention and treatment of AD is crucial to minimize the development of these potential complications (12).

The main strength of this study lies in its analysis of the relationship between adherence to the Mediterranean diet and allergic diseases in Korean adults using a large dataset collected by professionals. However, there are some limitations. First, the cross-sectional design prevents us from determining whether adherence to the Mediterranean diet causes or prevents allergic diseases, and future prospective studies are needed to confirm this. Second, while the Food Frequency Questionnaire (FFQ) used was self-reported and could be subject to recall bias, FFQs are widely accepted in epidemiological research for estimating dietary intake over time (27). Additionally, this study assessed the Mediterranean diet based solely on food frequency and quantity, without evaluating the role of micronutrients. Finally, without the use of biomarkers, we were unable to clarify the specific physiological mechanisms behind the Mediterranean diet’s impact on allergic diseases. Future research should consider including biomarkers to explore the mechanisms underlying the diet’s effects on allergic diseases in greater depth.

In summary, although this was a cross-sectional study, our findings revealed that adherence to the Mediterranean diet was negatively associated with the prevalence of AD in Korean adults, with this effect being more pronounced in women. Furthermore, among the components of the Mediterranean diet, fish and peanuts exhibited the most significant protective effects against AD and AR. Future prospective studies are necessary to validate these findings and to explore the mechanisms of the Mediterranean diet’s effects on allergic diseases, providing a stronger foundation for clinical practice.

## Data Availability

The datasets presented in this study can be found in online repositories. The names of the repository/repositories and accession number(s) can be found at: https://knhanes.kdca.go.kr/knhanes/main.do.

## Footnotes

^1^ https://knhanes.kdca.go.kr/knhanes/main.do
